# The removal of pluto from the class of planets and homosexuality from the class of psychiatric disorders: a comparison

**DOI:** 10.1186/1747-5341-7-4

**Published:** 2012-01-13

**Authors:** Peter Zachar, Kenneth S Kendler

**Affiliations:** 1 Department of Psychology, Auburn University Montgomery, 7061 Senators Drive, Montgomery, AL, 36117, USA; 2 Virginia Institute for Psychiatry and Behavioral Genetics and Departments of Psychiatry and Human Genetics, Medical College of Virginia of Virginia Commonwealth University, Virginia Commonwealth University Medical School, Box 980126, 800 E. Leigh Street, Room 1-123, Richmond, VA, USA 23298-0126

**Keywords:** classification, authority, objectivity, DSM

## Abstract

We compare astronomers' removal of Pluto from the listing of planets and psychiatrists' removal of homosexuality from the listing of mental disorders. Although the political maneuverings that emerged in both controversies are less than scientifically ideal, we argue that competition for "scientific authority" among competing groups is a normal part of scientific progress. In both cases, a complicated relationship between abstract constructs and evidence made the classification problem thorny.

## Introduction

The controversies over psychiatric classification in the past 30 years have garnered considerable attention. The existence of rancorous debates about how to classify is associated with claims that the developers of psychiatric diagnostic systems inappropriately clothe themselves in the aura of science without being scientific [1,2]. Although this article will not solve the problem of what counts as a "legitimate" scientific approach, it will make some claims about the role of debate between competing perspectives in the process of developing scientific classifications.

The purpose of the article is to draw comparisons between two different, yet surprisingly similar controversies, namely, whether Pluto is a planet and whether homosexuality is a psychiatric disorder. In our opinion a compelling argument can be made that Pluto never should have been classified as a planet to begin with and that homosexuality never should have been labeled a psychiatric disorder, and that the decisions to re-classify them were correct.

Let us discuss Pluto first. Pluto's existence and location was predicted by Percival Lowell based on discrepancies between the observed and predicted orbit for Uranus. Astronomers, however, made a mistake when predicting the orbit of Uranus. In their calculations they plugged in an incorrect size for Neptune's mass. If they had plugged in the correct size, the difference between the predicted and the observed orbits of Uranus would not have been so great [3]. It was mere accident that in 1930 Clyde Tombaugh, the discoverer of Pluto, found an object where Lowell said it should be.

With respect to homosexuality, in the late 19^th ^century there were active debates about whether same-sex attraction was a vice, a medical condition, or a harmless variation in behavior [4]. Its inclusion in psychiatric taxonomies was initially related to the scientifically discredited assumptions of degeneration theory - which began life as a theological concept but was naturalized following the introduction of evolutionary theories at mid-century [5,6]. During the heyday of degeneration theory, sexual practices such as masturbation and homosexuality were considered to be signs of a progressive psychic decline. Among the primary advocates of this view was the psychiatrist Richard von Krafft-Ebing, who was considered to be an authority on all manners of "perversion." In contrast, an opponent of the medicalization of homosexuality was Sigmund Freud - whose subsequent theories on the nature of perversions replaced those of Krafft-Ebing for most psychiatrists [7]. Neither the rejection of degeneration theory nor Freud's opposition, however, prevented homosexuality from being included in the first edition of the DSM.

An interesting similarity between the two cases was that many people in both disciplines had become committed to the classifications for a variety of reasons including respect for tradition and the role that the classification played in ongoing research programs - surely something that occurs in most fields. In both cases the constructs in question - planet and psychiatric disorder - were abstractions that grouped heterogeneous entities together. This allowed some debate about the proper scope of the construct, and about the relationship that pertains between new evidence and the classification.

A primary concern with this article is the issue of scientific authority. Where should authority reside if a scientific community cannot agree on how to resolve a controversy? This is an ongoing problem in psychiatry, as seen in disagreement between advocates for categorical and dimensional models and disagreements about what role biological substrates should play in psychiatric classification. We argue that if it is practical for the scientific community to allow disagreement and not force a decision, then it should. If that is not practical, then decisions are best made by a group of recognized and well-informed experts who consult widely. One of the most important aspects of such decisions, as illustrated by the Pluto and homosexuality controversies, is that members of the wider scientific community must perceive the process as fair. Fairness is an ethical concept. In particular, we will explore how voting can serve both ethical and epistemic ends in situations of uncertainty.

### The decision on Pluto: Not a planet

On August 24, 2006, members of the International Astronomical Union (IAU) voted to remove Pluto from the official list of planets. As noted earlier, Pluto was classified as a planet because of mistaken assumptions about its size at the time it was discovered. The event precipitating this reclassification was the discovery in 2005 of Eris, located beyond Pluto. Being larger than Pluto and having a moon, Eris appeared to be a 10^th ^planet.

Eris, however, has an orbit inclined 45 degrees relative to the orbital plane of Earth. All the planetary orbits are slightly inclined relative to Earth, but given its large inclination, some astronomers proposed that Eris does not belong in the class of things called planets because its orbital path is so different from that of the other planets The problem was that Pluto also has an inclined orbit, tilting 17 degrees relative to Earth's plane.

No definitive argument for keeping Pluto but excluding Eris from the set of planets existed. Why not allow Eris to be the 10^th ^planet? If Eris and Pluto were both classified as planets, several other bodies in the solar system would also have to be included. For example, in 1801, a large body between Mars and Jupiter called Ceres was discovered. It was briefly considered to be the 5^th ^planet. At that time astronomers believed that the gap between Mars and Jupiter was larger than it should be given the size of the gaps between the other planets. The location of Ceres made all the gaps systematic again. Soon thereafter another body called Pallas was discovered to also occupy the gap between Mars and Jupiter. Next Juno and Vesta were found. Rather than calling all of these bodies planets, they were classified as *asteroids*.

The discovery of Eris suggested to some that the status of Ceres should be revisited. Because Ceres can be differentiated from the other asteroids by being large enough to take a spherical shape, it was proposed that it could also be classified as a planet. One other complication is that like Ceres, Eris is a part of a larger group of objects. The group to which Eris belongs is called the Kuiper Belt. Many additional Kuiper Belt objects (KBOs) are larger than Pluto and have their own moons. A looser definition of planet that accepts both Eris and Ceres would also include many additional KBOs and swell the number of planets to over 20.

Pluto was a problematic case even before the discovery of Eris. In 2004 the IAU even created a Working Group on the Definition of a Planet to deal with the problem of Pluto's status [8]. After considering many proposals and counter-proposals, however, they could not come to consensus. A variety of factors contributed to this outcome. As Alan Boss [8] notes:

It is often said that the outcome of a committee's work is decided by who is chosen to sit on the committee. The same could be said of the IAU's Working Group on the Definition of a Planet (p. 120).

After the discovery of Eris, the chair of the Working Group forced a vote, in part because if the Working Group could not decide, the IAU Executive Committee might have resolved the problem on its own. It had to be resolved before Eris could be officially classified. Depending on the outcome, textbooks would have to be rewritten and expensive research programs such as the New Horizons mission would have to be redescribed. When the vote was taken, seven were in favor of keeping Pluto a planet, seven opposed, and seven favored a compromise that would make "planet" a superordinate category and then have several subcategories of planets.

There was considerable drama at the 2006 Prague conference where an official decision was to be made, including last minute changes in the proposal by a secretly formed Planet Definition Committee. The proposal that was accepted by a majority of the members present on the final day of the conference stated that a planet orbits a sun (is not a moon), is massive enough to take a spherical shape, and is not a member of a larger group of objects sharing the same orbital location (i.e., it has "cleared" the neighborhood around its orbit). Pluto, Eris and Ceres - which did not meet this definition - were put into a new category: *dwarf planets*.

### The decision on homosexuality: Not a psychiatric disorder

The construct of homosexuality has immense sociocultural significance. It has long been considered a perversion that is both immoral and illegal (see Leviticus 18:22 for male homosexuality). In modern astronomy, the scientific classification of the nine planets became a tradition to which people were committed. In psychiatry, commitment to a tradition preceded the classification, primarily because cultural, religious and legal prohibitions against homosexuality predated the birth of modern psychiatry.

On December 15, 1973 the Board of Trustees of the American Psychiatric Association (APA) voted to remove homosexuality from the official list of mental disorders. What precipitated this reclassification was not a single discovery; rather, it was a series of protests at the annual conventions of the American Psychiatric Association beginning in 1970. The protests initiated a scientific and professional debate by those who supported homosexuality's removal and those who wanted it to remain a disorder. Between 1970 and 1973, the debate was waged in the pages of psychiatry journals, in committee meetings, and psychiatric conferences.

Bayer's [9] listing of the relevant scientific information follows:

The human sexuality studies of Alfred Kinsey and his colleagues that showed surprisingly high prevalence rates (37%) for homosexual activity among males, suggesting that it may represent a normal variation in sexual behavior [10,11].

Anthropologist Clellan Ford and psychologist Frank Beach's [12] demonstration that prohibitions against homosexual behavior are not universal nor is the behavior limited to human beings.

Psychologist Evelyn Hooker's [13] findings that homosexual men are indistinguishable from a matched sample of heterosexual men with respect to psychopathology and that many homosexual parings can be classified as long-term and committed relationships rather than short term and compulsively driven relationships.

Two additional considerations also influenced psychiatrists to change their minds about homosexuality's classification. First, the protestors highlighted cases of social discrimination based on sexual orientation that were "justified" by claims that homosexuality was a mental disorder. This shocked some psychiatrists, who viewed their profession as playing a progressive role in defining homosexuality as a disorder rather than a moral failing subject to prosecution and imprisonment.

A second consideration was information gained from personal encounters with homosexuals, especially gay psychiatrists. Such encounters began to occur publically at the 1971 APA meeting. Research in social psychology has consistently demonstrated that positive personal encounters reduce negative attitudes about outgroups [14].

In one instance Robert Spitzer, who proposed the solution that was accepted by the Board of Trustees, attended a secret meeting of gay psychiatrists where he was able to hear their personal testimonies [9]. Spitzer [15] notes he came to see these individuals as underdogs and as being in pain, and decided that he wanted to help them. The DSM-II, which classified homosexuality as a sexual deviation, was prepared by the APA's Committee on Nomenclature and Statistics. The focus of the protestors thereby shifted to the Nomenclature Committee by late 1972 [9,16-18]. Those who supported classifying homosexuality as a disorder were concerned that, due to its composition, the Nomenclature Committee would support the deletion of homosexuality from the DSM-II [9]. In response they formed their own Ad Hoc Committee against the Deletion of Homosexuality and wanted the Nomenclature Committee's decision to be reviewed by a specially appointed committee more balanced in its composition. The astronomer Alan Boss's views about the effect that a committee's composition can have on the outcome were shared by psychiatrists some thirty years earlier.

As these debates continued, Spitzer came to the conclusion that homosexuality was different from other psychiatric disorders. He observed that in many cases it was accompanied by neither distress nor a general impairment in social functioning [19,20]. Many psychiatrists at the time held that heterosexual behavior represents optimal functioning and homosexual behavior suboptimal functioning. Spitzer [20] argued that if homosexuality was labeled a disorder because it is suboptimal, then other sub-optimal states should also be classified as mental disorders, including religious fanaticism, racism, and male chauvinism.

Unlike his opponents, Spitzer did not believe that his proposal would make it through the Nomenclature Committee, so he bypassed them [18,21]. He turned his focus to getting supportive votes from three additional committees, which paved the way for the official 1973 decision by the Board of Trustees.

### Opposition after the decision

One vocal opponent of the decision on Pluto was the geophysicist Alan Stern. After the vote, he said: "I am just disgusted by the way the IAU, which is meant to represent the best in science, handled this matter"[22] (p. 965). In collaboration with Mark Sykes, Stern circulated a petition stating that they do not agree with the new definition of a planet and would not use it. With over 300 signatories, a press release following the presentation of the petition stated:

Planning is underway to establish an open and inclusive grass-roots process by which planetary scientists and astronomers from around the world can approach a better resolution to the issue of planets in our solar system and elsewhere, with every step and discussion in public view. This process should culminate in a conference, not to determine a winner, but to acknowledge a consensus [23].

Sykes [24] has compared the vote to a papal decree, referring to the IAU classification as an ex cathedra listing of planets, which he calls *a misrepresentation of science as the promulgation of "truth" by an "authoritative" body*.

The procedural complaint that surfaced immediately following the decision was not that the definition was decided by a vote. If that had been the issue, the complaints would have preceded the vote. Prior to the conference when the proposition was that planets are massive enough to be spherical (which includes Pluto), Pluto's advocates saw a vote as representing informed scientific consensus. After the roundness definition was rejected, the complaint was that less than 30% of the IAU's 9000 members were at the conference and by the time the vote was taken on the conference's fourth day, only a few hundred attendees remained [25].

The controversy continued at a 2008 Conference titled *The Great Planet Debate: Science as Process*. The opponents of the new classification hoped that a different consensus would be forthcoming, but the outcome of the conference was quite similar to the outcome of the original Working Group. Although a trend was noted toward agreeing that the astronomical classifications of the future will have to account for more diversity then the current model's categories of planet, dwarf planet, asteroid, and comet, there was too much disagreement for any consensus to emerge.

One of the interesting differences between the cases in astronomy and psychiatry is that the official process for deciding an issue in psychiatry does not include a vote of the membership. Most of the authority is ceded to committees of experts.

According to Bayer [9] and Socarides [17,18], many psychiatrists vilified the decision on homosexuality as scientifically unsound, harmful to legitimate patients, immoral, politically motivated and a concession to the mob. Comparisons with dogmatic pronouncements of church councils were made as well. The difference between this case and that of Pluto was a sentiment among some conservative psychiatrists that not just the profession, but also morality and civilization itself, had been betrayed.

In essence, this movement within the American Psychiatric Association has accomplished what every other society, with rare exceptions, would have trembled to tamper with, a revision of a basic code and concept of life and biology [18] (p. 321)

After the vote of the Board of Trustees in 1973, the Ad Hoc Committee against the Deletion of Homosexuality did not go quietly away. Two of the chief opponents of the disclassification both prior to and after the vote were Socarides [17,26,27] and Bieber [28,29] who after the vote, circulated a petition, with 234 signatories, demanding a referendum of the membership on the status of homosexuality.

Like the astronomers in opposition to the Pluto decision, the protestors believed that the majority of the members would take their side. They used a policy that allowed a vote of the membership on procedural issues to force the Board to agree to a vote on the classification issue. Their argument was that the vote of the membership would reflect the scientific consensus, whereas the Board of Trustees' decision reflected political considerations.

The politics surrounding the vote were intense. The Ad Hoc Committee defended its claim that homosexuality was pathological with various psychoanalytic explanations. Those supporting the Board's decision argued that the substantial scientific issues had been decided by informed committees who studied the issues, and that the practice of choosing a scientific classification by a vote of members was inappropriate.

The vote of the membership was conducted by mail. The complaint in astronomy about the vote on the definition of planet was that it was limited to a small number of conference attendees. Many psychiatrists were concerned about a vote of the membership itself. In this vote, the decision of the Board was upheld by 58% of the voting members. Afterwards, the psychiatric opponents claimed that the vote did not reflect a scientific consensus because only 25% of the eligible voters turned in their ballots [18].

### Solving classification problems by proposing definitions

In both astronomy and psychiatry, the debates reached near crisis proportions. In both cases the solutions were proposed and voted on within a span of 12 months. In both cases controversy was fueled by considerable interest on the part of the media and the general public. For example, the relevant *New York Times *headline in each case read:

Psychiatrists in a Shift Declare Homosexuality No Mental Illness

Vote Makes It Official: Pluto Isn't What It Used To Be

The crisis nature of the Pluto and homosexuality decisions is signified by the pressure felt by the parties to reach a decision. In each case, the crisis was brought to closure by a proposed definition and agreement about how the definition was to be applied. This is more evident in the case of Pluto, where developing a definition was the explicit problem. In the case of homosexuality, Spitzer's compromise was also a definition, namely that a psychiatric disorder involves distress, social occupational dysfunction, or both. Once psychiatrists accepted the scientific data indicating that many cases of homosexuality involve neither subjective distress nor dysfunction, and accepted Spitzer's definition of disorder, they were able to agree on disclassification in spite of having different opinions about the desirability of variation in sexual orientation.

Couldn't astronomers have decided to not explicitly define a planet? Yes, they could have. If we list the physical properties of an object such as Pluto in terms of its composition, orbit, distance from the sun, evolution, etc., no one would add "planet" to that list. The construct of "planet" is physically irrelevant to the scientific study of Pluto. It does not matter to Pluto or Ceres whether they are classified as planets or dwarf planets.

The same cannot be said when we turn to classifying homosexuality as disordered or normal. Although any biological basis of a predisposition for homosexuality will not be altered by its being classified as a psychiatric disorder, the same cannot be said of its psychological manifestation. What homosexuality is like psychologically in an environment where it is considered a disorder will meaningfully differ from what it is like in an environment where it is considered a normal variation in sexual orientation.

An important difference between planetary astronomy and psychiatry is that, objectively speaking, there are typically fewer consequences if astronomers decide to leave a classificatory dilemma unsettled. Psychiatrists and psychologists do not always have that luxury. People present for treatment. Public health officials need information about risk factors and prevalence rates for specific conditions. Insurance companies require codes to process claims. Such consequences make it more difficult for psychiatrists to be what Mike Brown [30], the discoverer of Eris calls, "naive." By naive he means one can simply think about scientific considerations and not worry about the impact classifications have on culture.

### Scientific Authority

In both astronomy and psychiatry, advocates on both sides of the debate made claims that mirror the prototypical junk science rhetoric, namely, that the final decision (or opposition proposal) was not supported by rigorous scientific evidence, that the other side's acceptance reflected political rather than empirical considerations, and that the legitimate authority of "science" had been usurped. In both cases a large middle group avoided the political drama by not participating.

Astronomers disagreed about whether a planet can be defined in terms of inherent properties (roundness) or whether the role played by a body in a larger system is relevant (clearing one's orbit). Psychiatrists held different background assumptions about the validity of clinical experience versus empirical research. In each case, the groups in opposition were composed of people from different research traditions. In astronomy it was the geophysicists versus the dynamicists while in psychiatry it was practicing psychoanalysts versus research oriented academics.

How, though, are investigators to decide issues when the empirical evidence is deemed inadequate, and the slow process of filtering out the pretenders impractical? According to Beauchamp [31], when the evidence is inadequate, mature communities make decisions following procedures that are formulated to be fair. The role of procedure suggests that scientific authority does not lie with individual scientists, but with scientific communities - specifically in the publicly available writings produced by the communities over time. The scientific literature can be seen as a history of various research communities making competing claims [32,33]. Those claims that have survived the competitive process earn scientific authority. For example, in the debates between different research communities about the role of natural selection in evolution, the alternatives (the inheritance of acquired characteristics, mutationism, and orthogenesis) have been shown to not be viable, whereas natural selection is viable. The Darwinians' claims for the role of natural selection are therefore considered authoritative by the scientific community.

#### The role of the communities

Both Hull [32] and Longino [34] argue that the social nature of science, in which people inside and outside of a research community assess each other's work, is necessary for the objectivity on which scientific authority rests. Hull emphasizes the competitive nature of such assessment, a trend evident in both the Pluto and homosexuality controversies.

Although we claim that scientific authority belongs to communities, we do not seek to reify scientific authority and treat it as a concrete entity. Scientific authority is an idealized abstraction - analogous to abstractions like human rights and justice. No individual or committee can conform to this ideal for an extended period of time. As evident from the controversies herein described, those whose views do not prevail may experience disappointment - and may charge that the authorities who made the final decision betrayed scientific ideals.

An example of this disappointment is the complaints about deciding scientific matters by voting. A vote is a formal expression of opinion in response to a proposed decision. Obviously it is problematic for decisions on scientific matters to be subject to a timeline that is external to the accrual of adequate information, but is taking a vote inherently problematic? This view deserves scrutiny, primarily because procedures analogous to voting are accepted elsewhere in science, for example in grant and manuscript reviews. In situations of uncertantly where the evidence does not compel acceptance, voting has a role to play.

It is important to emphasize that rather than voting itself, what actually upset the critics in the Pluto and homosexuality controversies was that their belief that the wrong communities had been ceded authority. In astronomy, the geophysicists lost out to the dynamicists. In psychiatry, the old guard psychoanalysts lost out to younger, empirically-minded researchers.

Laudan [33] argues that conflicts between research traditions are a normal part of science. Various communities have different research problems and different stores of accessible information. When officially sanctioned classifications are being decided, however, the stakes become higher and the conflict intensifies. The allocation of authority to a single classification preempts the normal partitioning of epistemic authority across diverse sub-communities. Such forced decisions typically require compromises. But the expectation that the Platonic ideal called scientific authority can ever become fully incarnate probably fictional.

#### The role of experts

If community decisions are to rise above being a popularity contest they should be made by a group of informed experts. But who picks the experts? All communities have boundaries, defined by complicated transactions between in-group and out-group members. Decisions about who to include are always debatable.

In the controversies herein described, committees were selected by professional organizations who wielded political authority. There are also social structures in place that help to establish expertise. In theory, expertise represents a meritocracy where experts are identified with respect to their past achievements. In Hull's model, reputations are earned by proving oneself in the competitive process. "Reputation," while ideally about competence, also has a social element. For example, professional conferences function as occasions for networking and forming relationships. The relationships formed can contribute to the development of expertise.

In both astronomy and psychiatry, it was agreed that committee members should have knowledge and experience with respect to the classification issues under consideration, but the kind of knowledge they should possess was subject to debate. For example, in psychiatry one of the complaints about the Nomenclature Committee was that its members did not specialize in studying homosexuality. Many of the specialists, however, were invested in the construct's continued use and therefore biased with respect to the outcome (they did not want their specialty area eliminated). A broadly representative group may not be able to make a decision; a narrowly constructed group may be too partial.

Problems are inevitable when decisions about scientific classifications are subject to socially-enforced timelines. In agreement with the philosopher Miriam Solomon's [35] arguments, we hold that it would be better if empirical considerations were the leading factors in any "vote," but this is probably impossible. Even if a dispute could be reduced to "scientific issues" between research traditions, those in competing traditions have diverse interests and investments that are sure to influence their decisions. For these reasons, it would be naïve to believe that the answer to our classificatory dilemmas is always to go out and find more data. The problem is more typically one of deciding to which data (and experts) to give priority.

## Conclusion

Some readers of this history may conclude that the presence of partisanship and politics in astronomy does not justify partisanship and politics in psychiatric nosology; rather, in both cases it points to the fact that science is done by fallible human beings. However, conflict and controversy are also part of the rational process of scientific development.

Even if psychiatry is to be considered an immature science, one shouldn't conclude that the complicated dynamics of developing psychiatric classifications are solely contingent on either its immaturity or the unusual partisanship of its members. Any scientific discipline, no matter how well-developed, can be subject to such complications as long as (a) it relies on abstract constructs that classify a heterogeneous group as a single kind, (b) the classification problems have psychological, social or economic significance, and (c) the current classification does not satisfactorily account for all the data.

When such thorny classification problems occur, and their resolution cannot be delayed, disciplines need to a have a fair and systematic way of choosing "experts" to whom authority is given. Such groups need to be self-critical, consider multiple and conflicting perspectives, and not seek to merely defend their own strongly held positions. The *integrity *of this process - the choice of the experts and the approach of the experts to making decisions - does not assure agreement, but it is essential to the continued authority of scientific community itself.

## Competing interests

The authors report no competing interests. The authors alone are responsible for the content and the writing of the paper.

## Authors' contributions

The article was jointly written. Both authors have read and approve the final manuscript.
